# Changes in microbial community and enzyme activity in soil under continuous pepper cropping in response to *Trichoderma hamatum* MHT1134 application

**DOI:** 10.1038/s41598-021-00951-x

**Published:** 2021-11-03

**Authors:** Tingting Mao, Xuanli Jiang

**Affiliations:** 1 College of Agriculture, Guizhou University, Guiyang, People’s Republic of China; 2 Institute of Plant Protection, Guizhou Academy of Agricultural Sciences, Guiyang, People’s Republic of China

**Keywords:** Ecology, Environmental sciences, Microbiology, Applied microbiology, Microbial communities, Environmental microbiology, Fungi

## Abstract

To clarify the control effects of *Trichoderma hamatum* strain MHT1134 on *Fusarium* wilt in continuous pepper cropping fields and its regulatory effects on soil microecology, the physical and chemical properties, enzyme activities, community structures of soil samples from five field types were analysed. Samples were taken from fields that had been continuously planted for 1, 5, 9 years, and applied the strain MHT1134 for 1 and 2 years. The MHT1134 control effects on pepper wilt after application 1 year and 2 years were 63.03% and 70.21%, respectively. 4 kinds of physical and chemical indexes and 6 kinds of enzyme activities in soil were increased. With the continuous cropping years increased, the microbial abundance and diversity decreasing significantly. The relative abundances of *Fusarium*, *Gibberella* increased along with the planting years, but decreased after the MHT11134 application. However, the relative abundances of *Trichoderma* and *Chaetomium* significantly increased. Additionally, as the cropping years increased, the soil abundance of Actinobacteria gradually decreased, but it significantly increased from 17.56 to 22.44% after the MHT1134 application. Thus, strain MHT1134 effectively improved the microbial community structure of the soil, and it also positively affected soil quality. A continuous application may improve the control effect.

## Introduction

Pepper *Fusarium* wilt is an important and frequent disease, which is caused by *Fusarium oxysporum* Schlecht, in pepper (*Capsicum annuum* L.) production. Pepper wilt is a worldwide soil-borne disease, which pathogen have a wide host range and the spores can live in the soil for a long time^1^. It can be spread by rain and irrigation water, air currents, and seeds. When the conditions are suitable, the pathogen infects the wound or the intercellular space of the root system. Therefore, the occurrence of disease is mainly concentrated after transplanting or in the late growth period with abundant rainfall. It can multiply in the vascular bundles, causing vascular bundle blockage and preventing the plant from absorbing nutrients and water, thus causing the plant to wilt. Especially in continuous cropping fields, *F. oxysporum* can accumulate and spread in the field year after year^2^. Mao et al. reported that continuous cropping would aggravate the occurrence of pepper wilt disease, resulting in more than 80% yield loss in Guizhou Province, China^3^. It is difficult to control this soil-borne disease. At present, pepper *Fusarium* wilt is still mainly controlled by chemical fungicides. However, pesticide applications not only fail to control the disease effectively, they also destroy the soil microecology, inhibit the reproduction of beneficial microorganisms in the soil, and allow pathogenic microorganisms to evolve and multiply. The agricultural soil environment is polluted by residual harmful substances, which alter the balance of soil microbial population structures and negatively affect soil health^4^.

The vast majority of biological control methods are environmentally friendly and can overcome the disadvantages caused by pesticides. In particular, the exploitation and utilisation of microbial resources have become more and more important in plant disease control^5^. For instance, antagonistic fungi, such as *Trichoderma harzianum* strain CH1, *Trichoderma asperellum* strain MC1, *Trichoderma brevicompactum* MF1^6^, *Chaetomium globosum* LJ-S2L1^3^ and the mutant of *Trichoderma viride* TvM2-UV/60 show activities against *F. oxysporum*^7^. Additionally, some antagonistic bacteria, such as isolates BR6 and BR9^8^, *Bacillus* sp. MB015 and *Pseudomonas* sp. MB108^9^ have obvious inhibitory effects on *F. oxysporum*. *Bacillus subtilis* and *Pseudomonas fluorescens* show superior inhibitory effects against *F. oxysporum* growth^10^. Moreover, many biocontrol agents regulate microbial community structures when applied to soil. Zhong et al*.*^11^ found that applying bio-organic fertilizer for two consecutive years not only significantly reduces the *F. oxysporum* level and makes the soil culturable microbial community structure more balanced, but it also improves yield and fruit quality of banana. Shen et al*.*^12^ found that by applying microbial agents for two consecutive years, soil bacterial diversity and microbial community structure became optimised, thus reducing the plant disease incidence. At present, research on pepper *Fusarium* wilt mainly focuses on the differences in soil microbial community diversity between diseased and healthy plants. There are few studies on the changes in soil microecology, the physical and chemical properties, and enzyme activity levels after the application of biocontrol agents in continuous cropping fields. MHT1134 strain is a biocontrol fungus screened by us in the early stage. We have previously reported on the biocontrol and growth-promoting effects of this strain. The strain can not only achieve 81.80% inhibition of *F. oxysporum*, but also has good inhibitory effects on seven other pathogens. In addition, the application of the MHT1134 fermentation broth increases the pepper yield after 1 year^13^. The Illumina MiSeq 2500 high-throughput sequencing platform was used to study the effects of a continuous 2 years application (twice application per year) of the biocontrol fungal strain MHT1134 fermentation broth on the microbial community structure and physicochemical properties of pepper rhizosphere soils in continuous cropping fields. The aim is to explore the soil microbiological mechanisms used by the biocontrol fungal MHT1134 against pepper *Fusarium* wilt.

## Materials and methods

### Fungal strain and fermentation broth preparation

*Trichoderma hamatum* strain MHT1134 was originally isolated from rhizosphere soil of pepper^13^ by Mao and colleagues from Guizhou University and Guizhou Institute of Plant Protection, China. It was stored in the China Center for Typical Microbiology (CCTCC 2018709). To prepare the MHT1134 fermented broth, the strain was inoculated on potato dextrose agar (PDA) plates in the dark at 28 °C for 7 days. Then, the culture was made into cakes and inoculated into potato dextrose (PD) liquid medium with shaking at 160 rpm and 25 °C for 7 days. Afterward, the inoculum was filtered using double-layered gauze. To increase the conidia content of the MHT1134 fermented broth, it was allowed to grow for another 5 days. Finally, Sterilized PD liquid was added to adjust the inoculation density to 1 × 10^6^ cfu/mL.

### Profile of the test site and experimental design

Field tests were conducted in pepper base from May 2017 to November 2017 and from May 2018 to November 2018. The base is a test site of Institute of Plant Protection, Guizhou Academy of Agricultural Sciences, China, which is specially used for experimental research, and we conducted field experiments in this test site. The base has a total of 34 hectares, where soil type was yellow, and the climate was subtropical humid monsoon. The average temperature in the hottest month (July) was 20.7 °C. The variety of pepper grown in this base is dafang knit pepper, which is a local conventional variety produced and supplied by a company named Juli. The experimental site was located in the pepper planting base of Huangnitang Town, Dafang County, Bijie City, Guizhou Province, China (27° 10′ 24″ N, 105° 42′ 17″ E, elevation 1314.0 ± 3 m). There were three kinds of fields planted for 1 year, 5 years and 9 years respectively in the base, and the pepper wilt disease occurred in the field continuously cultivated for 5 years and continuously cultivated for 9 years. We tested three different types of fields with similar locations, the same soil quality and topography. The collection of plant material has been approved by Institute of Plant Protection, Guizhou Academy of Agricultural Sciences, Guiyang City, P. R. China. Experimental research and field study on pepper in this study has complied with the IUCN Policy Statement on Research Involving Species at Risk of Extinction.

Our experiment was based on the premise that the pepper wilt disease occurred naturally in the field. There were five treatments in the experiment, respectively. Plots undergoing pepper cultivation for 1 year, which was uncultivated wasteland before the experiment (CC1); plots undergoing continuous pepper cultivation for 5 years (CC5); plots undergoing continuous pepper cultivation for 9 years (CC9); plots were randomly allocated 1 year in advance to apply MHT1134 fermentation broth for 1 year in pepper fields that had been continuously cropped for 9 years (TR1). Plots were randomly allocated 2 years in advance to apply MHT1134 fermentation broth for 2 year in pepper fields that had been continuously cropped for 9 years (TR2). Each treatment was set up with 3 repeating plots, there are a total of 15 experimental plots, each plot was 80 m^2^, and 308 pepper plants were planted. The plant distance between each pepper plant was 30 cm. The spacing is 80 cm.

### Application of MHT1134 fermentation broth

The inocula was prepared in accordance with the preparation method of strain MHT1134^13^. During the transplanting of pepper seedlings (May 10, 2017 and May 6, 2018), in the plots of TR1 and TR2, 50 mL MHT1134 fermentation broth containing spores was irrigated into the hole dug for each plant, covered with a little soil, and then the pepper seedlings were transplanted. Before the pepper flowering period (July 9, 2017 and July 5, 2018), 50 mL of MHT1134 strain fermentation broth containing spores at a concentration of 1 × 10^6^ cfu/mL was irrigated into the pepper rhizosphere again. The peppers in the plots of CC1, CC5 and CC9 were irrigated with medium without spores of strain MHT1134 for a year (twice per year). And CC9 treatment was used as the control without biocontrol under the same conditions.

### Control effect investigation

Pepper plants with typical wilt symptoms were collected from plots treated with CC9, TR1 and TR2. The pathogen was isolated from the brown tissues of roots by tissue separation^14^. The strain were identified by microscopic morphology observation and DNA extraction comparison, for specific methods, refer to the method in the article published by Mao et al.^13^*.* Then, spore suspension with concentration of 1 × 10^6^ cfu/mL was prepared according to the method of *Fungal strain and fermentation broth preparation*. 10 mL spore suspension was injected into the root of pepper seedlings for inoculation, and the pepper plants were cultured in a climate incubator at 25 °C and 90% relative humidity. Investigation began after 5 days. Once the pepper showed the same symptoms, it was pulled out and the tissue was scraped from the root and stem for observation, to confirm whether it was the same with the inoculated pathogen.

After identifying the species of *Fusarium* wilt in the field, the incidence of *Fusarium* wilt in each treatment was investigated. Investigation begins at the onset of wilt disease in the field and continues until the pepper harvest. The number of diseased plants at different levels was recorded in the period of pepper engraftment, flowering and fruiting. The Percent Disease Index (PDI) and control effect were calculated after integrated statistics. The survey methods were published previously in Mao et al.^13^.

### Soil sample collection

Based on the reported colonization rule of *Trichoderma* in the soil^15^, Five treated soil samples were collected on day 60 (September 3, 2018), day 90 (October 3, 2018) and day 120 (November 2, 2018) after the second biocontrol strain application. The soil samples from each stage of each treatment were mixed into one sample for the next study. The five-point sampling method was used to take the soil from approximately 10 cm below the rhizosphere soil of the pepper, and the rhizosphere soil of three pepper trees was taken from each point. The collected rhizosphere soil of each plot was mixed evenly as a sub-sample and taken to the laboratory for preservation at − 80 °C.

### Soil physical and chemical properties

Soil samples from five different treatments were collected for physical and chemical property detection. Alkali-hydrolysable nitrogen levels were determined using the alkali-hydrolysable diffusion method^16^. Soil available phosphorus and potassium levels were determined by the sodium bicarbonate extraction of molybdenum and antimony anticolourimetry and by ammonium acetate extraction of flame photometry, respectively^17^. The organic matter content was determined using the potassium dichromate volumetric method^18^.

### Enzymatic activity

The urease, dehydrogenase, invertase, acid phosphatase, catalase and acid protease activities in the pepper soil were measured. The soil urease activity was determined using indophenol blue colorimetry, and the acid phosphatase activity was determined using the disodium phenyl phosphate colorimetry method. The catalase activity was determined using the KMnO_4_ titration method, invertase activity was determined using 3,5-dinitrosalicylic acid colorimetry, and both dehydrogenase and acid protease activities were determined using the double-antibody sandwich method^19^.

### Analysis of the microbial community

Microbial community genomic DNA was extracted from each of the samples using an E.Z.N.A.^®^ soil DNA Kit (Omega Bio-tek, Norcross, GA, USA) following the manufacturer’s recommendations. The DNA extract was checked on a 1% agarose gel, and DNA concentration and purity were determined using a NanoDrop 2000 UV–Vis spectrophotometer (Thermo Scientific, Wilmington, DE, USA).

The hypervariable region V3–V4 of the bacterial 16S rRNA gene were amplified with the primer pair 338F/806R^20^ on an ABI GeneAmp^®^ 9700 PCR thermocycler (Applied Biosystems, Foster City, CA, USA). The ITS regions of fungi were used as the target sequences. The PCR amplification was carried out with the universal primers ITS1F and ITS2R^1^. PCR reactions were performed in triplicate. The PCR product was extracted from a 2% agarose gel and purified using the AxyPrep DNA Gel Extraction Kit (Axygen Biosciences, Union City, CA, USA) in accordance with the manufacturer’s instructions and quantified using Quantus™ Fluorometer (Promega, USA). Finally, the Illumina paired-end library preparation, cluster generation and Illumina MiSeq PE300 paired-end sequencing were performed by Majorbio Bio-Pharm Technology Co. Ltd. (Shanghai, China).

The raw gene sequencing reads were demultiplexed, quality-filtered by fastp version 0.20.0^21^ and merged using FLASH version 1.2.7^22^. Operational taxonomic units (OTUs) with 97% similarity cut-offs were clustered using UPARSE version 7.1^23,24^, and chimeric sequences were identified and removed. The taxonomy of each OTU representative sequence was analysed by RDP Classifier version 2.2^25^ against the DNA database using a confidence threshold of 0.7.

### Statistical analyses

The experiments were organised in a completely randomised design. Three replicates were set for each treatment. Data were subjected to an analysis of variance using SPSS sofware (IBM Corporation, Armonk, NY, USA). Treatment means were separated using Duncan’s multiple range test (P < 0.05 and P < 0.01).

## Results

### Field control effect of strain MHT1134 on Fusarium wilt of pepper

**Figure 1:**
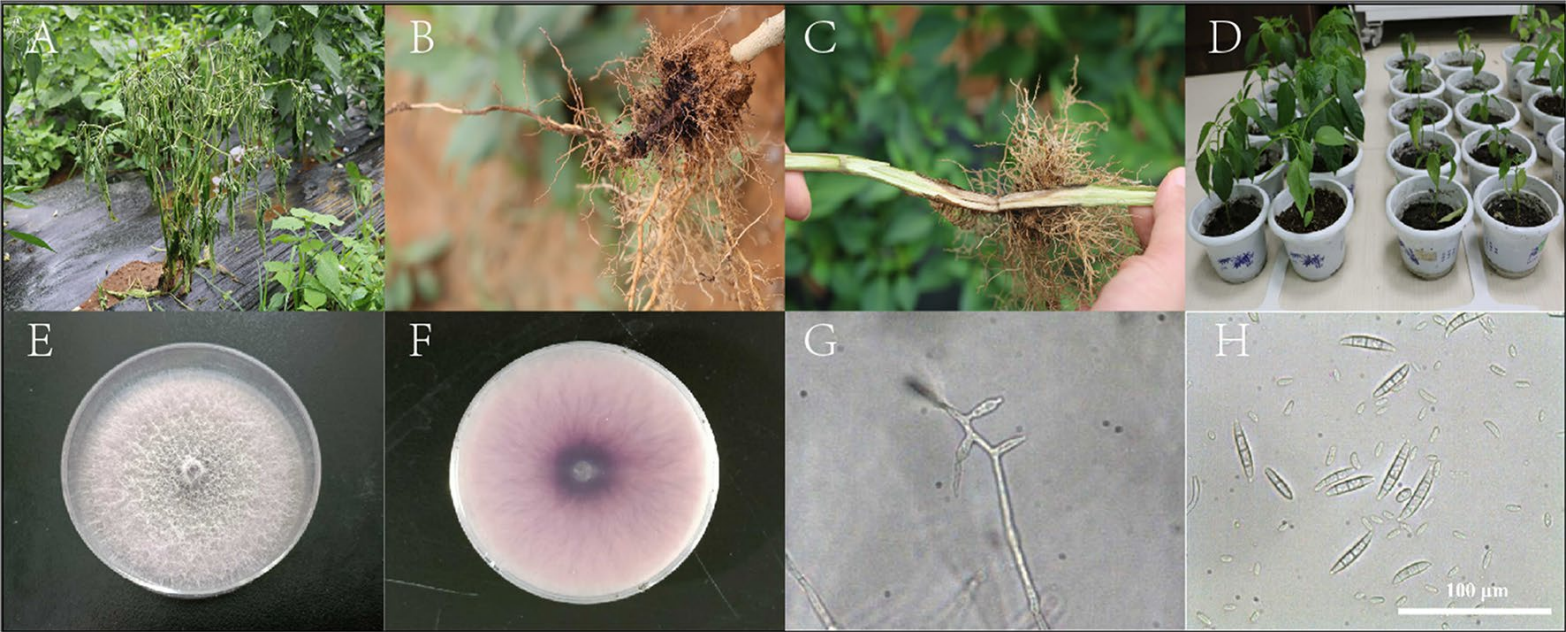
Typical symptoms and identification of pathogen strains of pepper *Fusarium* wilt in experimental sites. (**A**) At the late stage of *Fusarium* wilt, the whole plant withered and died; (**B**) the lateral root and taproot of the pepper turn brown and rot; (**C**) discoloration of vascular bundle in pepper stem after cutting; (**D**) after the isolated *F. oxysporum* was inoculated on the pepper, which showed the initial symptoms of wilt disease; (**E**) positive characteristics of *F. oxysporum* colony; (**F**) negative characteristics of colony; (**G**) sporulation peduncle in bottle shape; (**H**) large and small conidia.

Before the investigation of strain MHT1134 control effect, pepper plants with the same wilt symptoms were collected from CC9, TR1 and TR2 fields. The same wilt symptom is that the lower leaves of the plant turn yellow or fall off, and the whole seedling plant wilt and die in the later stage. The pepper root neck can be seen with obvious water-stained brown disease spots. When the root and stem are cut open, the vascular bundle turns brown and has a trend of upward stretching (Fig. 1A–C). We isolated a strain in the root, which colony color is purple (Fig. 1E,F), On the sixth day after inoculating healthy pepper with the spore suspension, the plants showed lower leaf shedding and plant wilting (Fig. 1D). And the pathogen was isolated in the root with the same colony characteristics and micromorphology. The main classification features are as follows: the conidiophores are colorless, with bottle-shaped spore-producing cells at the top (Fig. 1G). There are two kinds of conidias. The small conidia are monocytic, oval or kidney shaped, colorless and are 5–12 × 2–3.5 μm in size. Large conidia are multicellular, sickle-shaped, slightly curved, with slightly pointed cells at both ends, colorless and are 19.6–39.4 × 3.5–5.0 μm in size (Fig. 1H). The morphological characteristics of the strain were consistent with *Fusarium oxysporum*. The strain DNA was extracted and ITS sequence was amplified by PCR to obtain a DNA fragment with a length of about 500 bp. The sequencing results were compared with the gene sequences in Genbank, and the highest homology was found in *Fusarium*, and the sequence homology with *Fusarium oxysporum* reached 100%. The pathogen of pepper wilt was *Fusarium oxysporum* by means of morphological and molecular identification.

Compared with CC9 treatment without biocontrol fungi MHT1134, the disease rate and disease index of pepper *Fusarium* wilt in TR1 and TR2 treatment were decreased. In TR1, the disease rate and disease index of pepper wilt decreased by 8.44% and 3.76%, respectively. In TR2, the disease rate and disease index of pepper wilt decreased by 57.69% and 63.02%, respectively. However, in the TR2 plots over 2018 and 2019, the disease rate and disease index decreased to 7.13% and 3.03%, which were 64.26% and 70.20%, respectively, less than in the CC9 plots. The control effect of MHT11341 on pepper wilt was 63.03% and 70.21% after one and two years of continuous cropping field, respectively (Table 1). The results indicated that the continuous application of a biocontrol strain further consolidated and improved the control effect.

**Table 1:** Control effects of strain MHT1134 on *Fusarium* wilt in continuous pepper cropping fields.

Treatment	Disease incidence (%)	Disease index	Control efficacy (%)
MHT1134 was applied for 1 year	8.44 ± 0.27b	3.76 ± 0.20b	63.03 ± 1.99b (A)
MHT1134 was applied for 2 year	7.13 ± 0.46b	3.03 ± 0.10b	70.21 ± 1.03a (A)
Continuous cropping 9 years without MHT1134	19.95 ± 0.40a	10.17 ± 0.10a	–

The data are mean ± SD. The different capital and lowercase letters in the same column indicate significant different at P < 0.1 and P < 0.05 level by Duncan’s new multiple range test.

### Effects of strain MHT1134 on the physical and chemical properties of pepper rhizosphere soil

Soil samples from different planting years showed differences in their physical and chemical properties. In particular, the contents of available phosphorus, available potassium and organic matter were significantly different between the soil planted for the first year and the soil continuously planted for 9 years (available phosphorus: F = 4.38 p = 0.03; available potassium: F = 2.94 p = 0.009; organic matter: F = 5.45 p = 0.02). With the increase in planting years, the organic matter and alkali-hydrolysable nitrogen contents in the soil showed decreasing trends. The organic matter content in the CC9 soil samples was 23.64% less than in the CC1 soil samples, and the alkali-hydrolysable nitrogen content was 45.2% less. The available phosphorus and available potassium levels did not show regular change trends, but the available potassium content in the CC9 soil was lower than in the CC1 soil.

Compared with the CC9 soil samples, the alkali-hydrolysed nitrogen, organic matter, available phosphorus and available potassium contents in TR1 soil samples increased by 46.82%, 6.26%, 5.09% and 47.06%, respectively. The available potassium content increased most obviously, followed by alkali-hydrolysable nitrogen. The alkali-hydrolysable nitrogen, organic matter and available phosphorus contents decreased slightly in TR2, but were still higher than those in the CC9 soil samples. In addition, the available potassium content continued to increase by 20% after the application of biocontrol bacterium MHT1134 in the second year (Table 2).

**Table 2:** Effects of MHT1134 on physical and chemical properties of the pepper rhizosphere soil.

Treatment	Organic matter content/(g/kg)	Alkaline hydrolysis nitrogen content/(mg/kg)	Available phosphorus content/(mg/kg)	Available potassium content/(mg/kg)
CC1	26.57 ± 30.79a	304.63 ± 17.28a	26.69 ± 7.65d	0.35 ± 0.02a
CC5	22.85 ± 2.46b	172.45 ± 84.05cd	46.79 ± 5.70a	0.35 ± 0.04a
CC9	20.29 ± 1.79d	166.91 ± 38.70d	29.67 ± 4.77bc	0.17 ± 0.08d
TR1	21.56 ± 0.56c	245.05 ± 73.76b	31.18 ± 1.03b	0.25 ± 0.05c
TR2	21.28 ± 0.57c	184.45 ± 32.44c	28.44 ± 3.53bc	0.30 ± 0.05b

CC1, CC5 and CC9, represent the plots where pepper had been continuously planted for 1, 5 and 9 years, respectively, and TR1 and TR2 represent CC9 plots in which the MHT1134 biocontrol fermentation broth had been applied 1 and 2 years in advance, respectively. The data are mean ± SD. The different lowercase letters in the same column indicate significant different at P < 0.05 level by Duncan’s new multiple range test.

### Effects of strain MHT1134 on enzymatic activities in pepper rhizosphere soil

**Figure 2:**
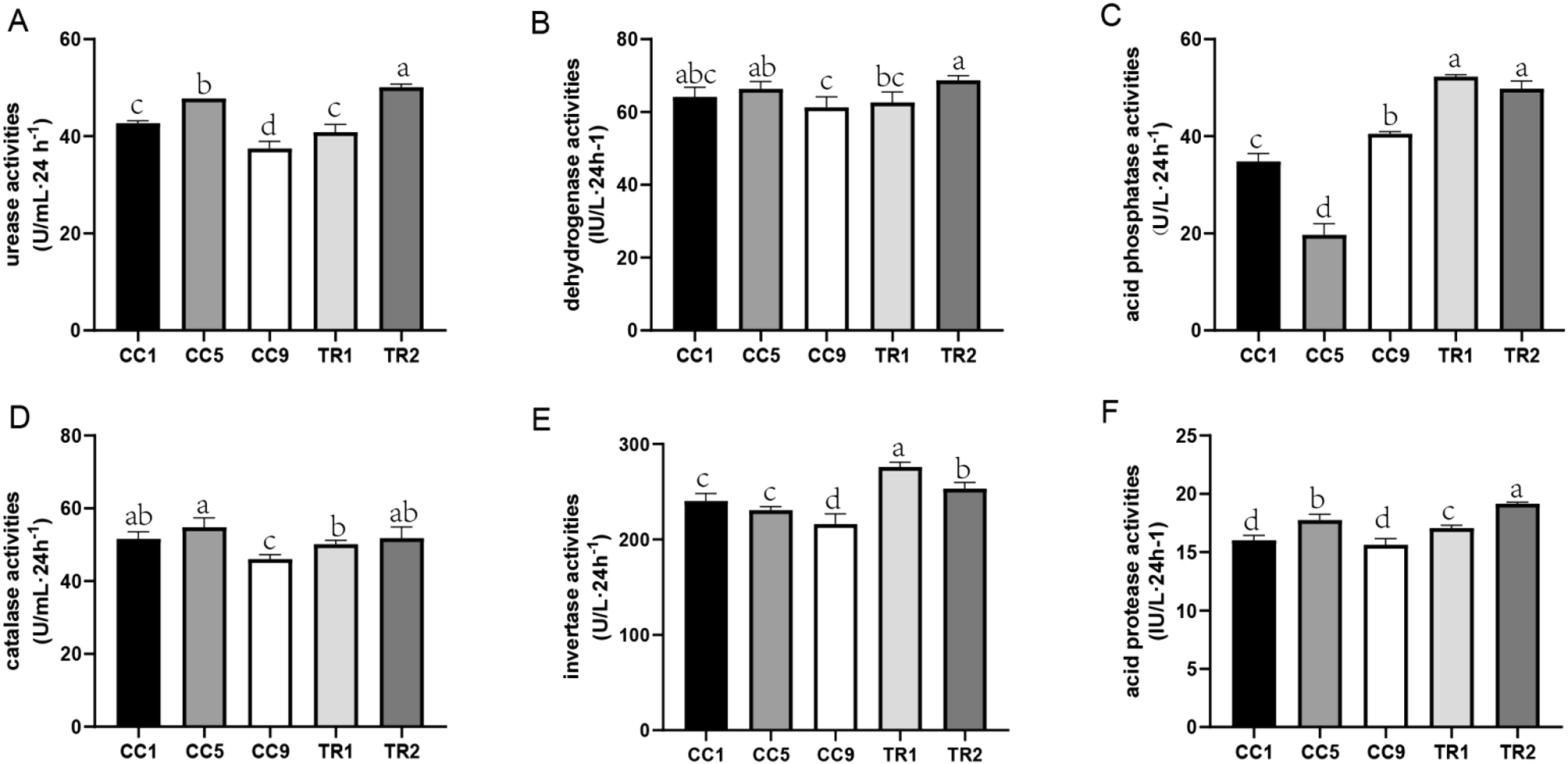
Differences in the enzyme activities in the continuously cropped pepper rhizosphere soil after the application of strain MHT1134. Activity levels of (**A**) urease; (**B**) dehydrogenase; (**C**) acid phosphatase; (**D**) catalase; (**E**) invertase; and (**F**) acid protease. CC1, CC5 and CC9, represent the plots where pepper had been continuously planted for 1, 5 and 9 years, respectively, and TR1 and TR2 represent CC9 plots in which the MHT1134 biocontrol fermentation broth had been applied 1 and 2 years in advance, respectively.

By comparing the activities of six kinds of enzymes in the five groups of soil samples, we found that all the activities, except for that of acid phosphatase, in the CC9 soil were lower than those in the CC1 soil. In TR1 and TR2, the activities of the six enzymes in the soil increased. The urease, dehydrogenase, acid phosphatase, catalase, invertase and acid protease activities increased by 9.04%, 4.42%, 29.02%, 9.35%, 17.83% and 6.83% in TR1, respectively, and by 18.60%, 20.26%, 22.86%, 18.87%, 16.59% and 14.30% in TR2, respectively (Fig. 2A–F). The results indicated that MHT1134 applications could improve the enzyme activities in the soil to different degrees. Moreover, the urease, dehydrogenase, catalase and acid protease activities in soil significantly increased after the continuous application of MHT1134.

### Microbial diversity and richness

The sample dilution curve tended to be flat, and the fungal and bacterial diversity index table (Table 3) shows that the library coverage levels were greater than 99% and 98%, respectively. Together, they indicate that the OTU coverage of the soil samples is basically saturated; therefore, the OTUs reflect the species and structures of the fungal and bacterial communities in the samples. High-throughput sequencing results showed that 765,747 16S rRNA sequences and 1,012,237 ITS sequences were obtained from 15 samples of pepper rhizosphere soil subjected to five treatments. After data quality control, there were 35,362–72,498 bacterial 16S rRNA sequences and 54,007–74,562 fungal ITS sequences. In addition, using the 97% standard, the bacterial and fungal OTU numbers were 17,444–47,775 and 50,876–71,236, respectively.

**Table 3:** Alpha-diversity indexes of fungi and bacteria in different continuous pepper cropping soils.

	**Treatments**	**Sobs**	**Shannon**	**Simpson**	**Ace**	**Chao**	**Coverage**
Fungi	CC1	127.67 ± 4.78b	2.88 ± 0.13a	0.12 ± 0.02a	129.46 ± 4.27b	135.70 ± 4.48b	0.99 ± 0.00a
	CC5	127.00 ± 5.72b	2.69 ± 0.19c	0.13 ± 0.02b	130.70 ± 5.47c	130.90 ± 4.70c	0.99 ± 0.00a
	CC9	134.33 ± 3.86a	2.71 ± 0.16a	0.13 ± 0.02a	141.99 ± 2.05c	142.37 ± 2.58b	0.99 ± 0.00a
	TR1	136.33 ± 7.04c	2.72 ± 0.27ab	0.15 ± 0.02b	138.99 ± 4.76b	142.23 ± 1.52b	0.99 ± 0.00a
	TR2	134.67 ± 4.99ab	2.91 ± 0.24c	0.13 ± 0.04a	137.8 ± 5.05a	137.45 ± 4.56a	0.99 ± 0.00a
Bacteria	CC1	2083.67 ± 125.99	6.62 ± 0.05	2.94 ± 0.02	2319.21 ± 130.03	2364.43 ± 123.51	0.99 ± 0.00a
	CC5	1850.33 ± 125.39	6.59 ± 0.03	2.72 ± 0.1	2047.83 ± 160.36	2070.13 ± 150.62	0.99 ± 0.00a
	CC9	1843.00 ± 71.01	6.57 ± 0.06	3.26 ± 0.8	2090.87 ± 66.11	2113.15 ± 86.55	0.98 ± 0.00a
	TR1	1667.67 ± 99.72	6.24 ± 0.09	4.69 ± 0.58	1906.17 ± 134.27	1931.39 ± 132.11	0.99 ± 0.00a
	TR2	1809.00 ± 157.92	6.17 ± 0.18	6.04 ± 0.12	2031.43 ± 170.75	2059.70 ± 159.25	0.99 ± 0.00a

CC1, CC5 and CC9, represent the plots where pepper had been continuously planted for 1, 5 and 9 years, respectively, and TR1 and TR2 represent CC9 plots in which the MHT1134 biocontrol fermentation broth had been applied 1 and 2 years in advance, respectively. The data are mean ± SD. The different lowercase letters in the same column indicate significant different at P < 0.05 level by Duncan’s new multiple range test.

### Alpha-diversity analysis of fungi and bacteria

The changes in fungal and bacteria diversity are shown in Table 3. According to the Shannon index analysis, the species richness of fungi in CC1 was the highest (2.88). As the planting years increased, the Shannon index decreased gradually (2.71 in CC5 and 2.69 in CC9). Although ACE and Chao indexes, representing the species abundance of the community, did not show obvious increasing trends, in CC9, the values of the two indexes were significantly higher than in CC1, which indicated that as the planting years increased, the diversity of fungi in the pepper soil decreased, while the species abundance increased. As shown in Table 3, in TR1, the Simpson index, representing species dominance, and the Sobs index, representing species richness, increased significantly, and the Shannon index also increased. In TR2, the Shannon index increased significantly, while the values of other indexes decreased slightly. We hypothesised that after the first year of application, the strain MHT1134 colonised in large numbers, resulting in it being the dominant community species. After continuous application, the soil ecology had adjusted, and the diversity of soil fungi continued to increase. In general, the application of the biocontrol fungal MHT1134 increased the diversity of fungi in the pepper rhizosphere soil and decreased the dominance of some species.

The changes in bacterial diversity and abundance in the pepper rhizosphere soil after different periods of continuous cropping are shown by the decreases in the Shannon and Sobs indexes decreased as the planting years increased, indicating that bacterial diversity and bacterial community richness decreased. Although ACE and Chao indexes representing the species abundance of the community did not show regular decreasing trends, in CC9, the values of the two indexes were significantly lower than in CC1, indicating that as the planting years increased, the diversity and richness of bacteria in the pepper soil decreased. Strain MHT1134 had no significant effect on the alpha-diversity index of soil bacteria in TR1, but Simpson, ACE and Chao indexes increased in TR2.

### Effects of MHT1134 on the microbial community structure in pepper rhizosphere soil

All the bacteria were classified into 352 genera and 23 phyla according to their 16S rRNA sequences, and all the fungi were classified into 6 phyla and 194 genera according to their ITS sequences. The top five phyla in terms of bacterial abundance were Actinobacteria, Acidobacteria, Chloroflexi, Gemmatimonadetes and Nitrospirae. The top six phyla in terms of fungal abundance were Ascomycota, Zygomycota, Basidiomycota, Glomeromycota, Chytridiomycota and Rozellomycota.

### Effects of MHT1134 on fungal community structure in pepper rhizosphere soil

**Figure 3:**
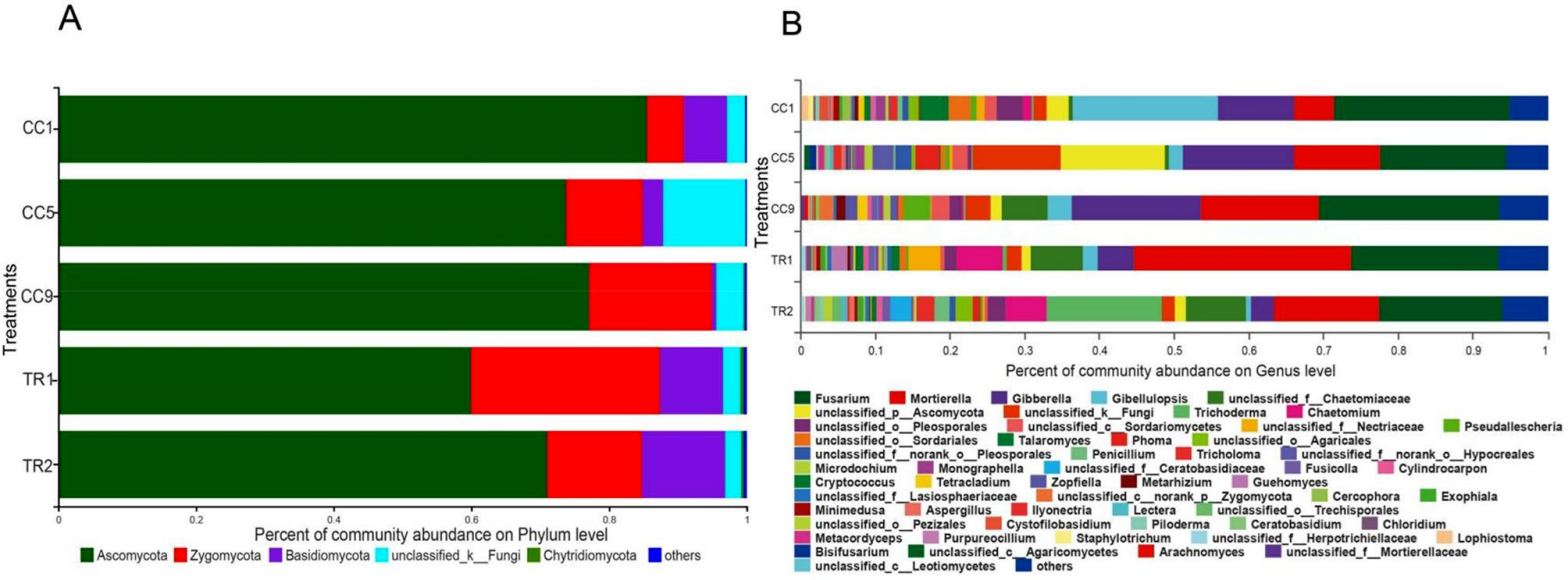
Fungal clustering accumulation map in pepper rhizosphere soil at the phylum (**A**) and genus (**B**) levels. CC1, CC5 and CC9, represent the plots where pepper had been continuously planted for 1, 5 and 9 years, respectively, and TR1 and TR2 represent CC9 plots in which the MHT1134 biocontrol fermentation broth had been applied 1 and 2 years in advance, respectively.

The effects of the biocontrol treatment on fungal phyla are shown in Fig. 3A. After treatment with MHT1134, the relative abundance of Ascomycota decreased significantly from 77.9 to 70.99%. The abundance of Basidiomycota increased significantly after the treatment, whereas it decreased with the continuous cropping time before the MHT1134 application. However, Zygomycota increased in abundance with the continuous cropping time. The abundance of strain MHT1134 increased significantly and then decreased by 1 year after treatment.

By analysing the relative abundance of fungi of different genera in the soil, it was found that the fungi of several genera showed similar change trends in different soil treatments. The relative abundances of *Fusarium*, *Gibberella* and the alkali-resistant fungus *Pseudallescheria* in the soil increased along with continuous cultivation years (CC1 < CC5 < CC9). The relative abundance levels of fungi in these three genera decreased in TR1 and TR2 (CC9 > TR1 > TR2). In addition, the trend was found for *Trichoderma*, *Chaetomium* and *Mortierella*, which declined as the planting years increased, but their relative abundance levels significantly increased in TR1 and significantly increased again in TR2 (Fig. 3B).

**Figure 4:**
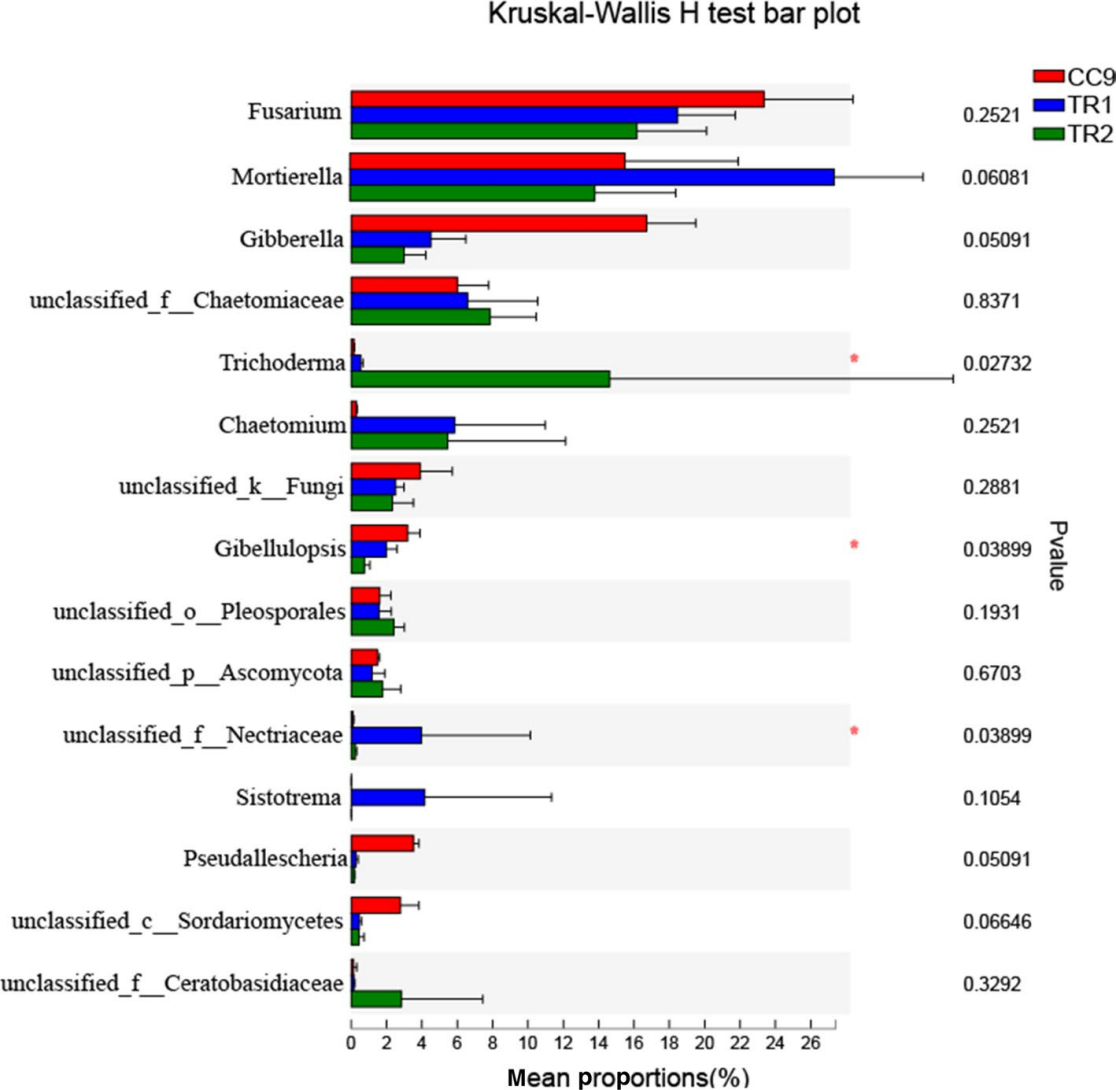
The relative abundances of the first 15 genera after the MHT1134 application. *0.01 < *P* ≤ 0.05. CC9 represent the plots where pepper had been continuously planted for 9 years, and TR1 and TR2 represent CC9 plots in which the MHT1134 biocontrol fermentation broth had been applied 1 and 2 years in advance, respectively.

Using *Fusarium* as the control, we analysed the variation trends of microorganisms in CC9, TR1 and TR2 soil samples. As shown in Fig. 4, the levels of three genera were positively correlated with the *Fusarium* change trend, *Gibellulopsis*, *Giberella* and *Pseudallescheria*, while three genera, *Trichoderma*, *Chaetomium* and *Mortierella*, were negatively correlated with *Fusarium*. Thus, the abundance levels of fungi in *Gibellulopsis*, *Gibberella* and *Pseudallescheria* were reduced after the MHT1134 application. Some species of *Gibellulopsis* are the pathogenic fungi that cause *Verticillium* wilt, and some species of *Gibberella* are the pathogenic fungi that cause gibberellic diseases. The abundance levels of *Trichoderma*, *Chaetomium* and *Mortierella* significantly increased after the application of strain MHT1134.

### Effects of MHT1134 on bacterial community structure in pepper rhizosphere soil

**Figure 5:**
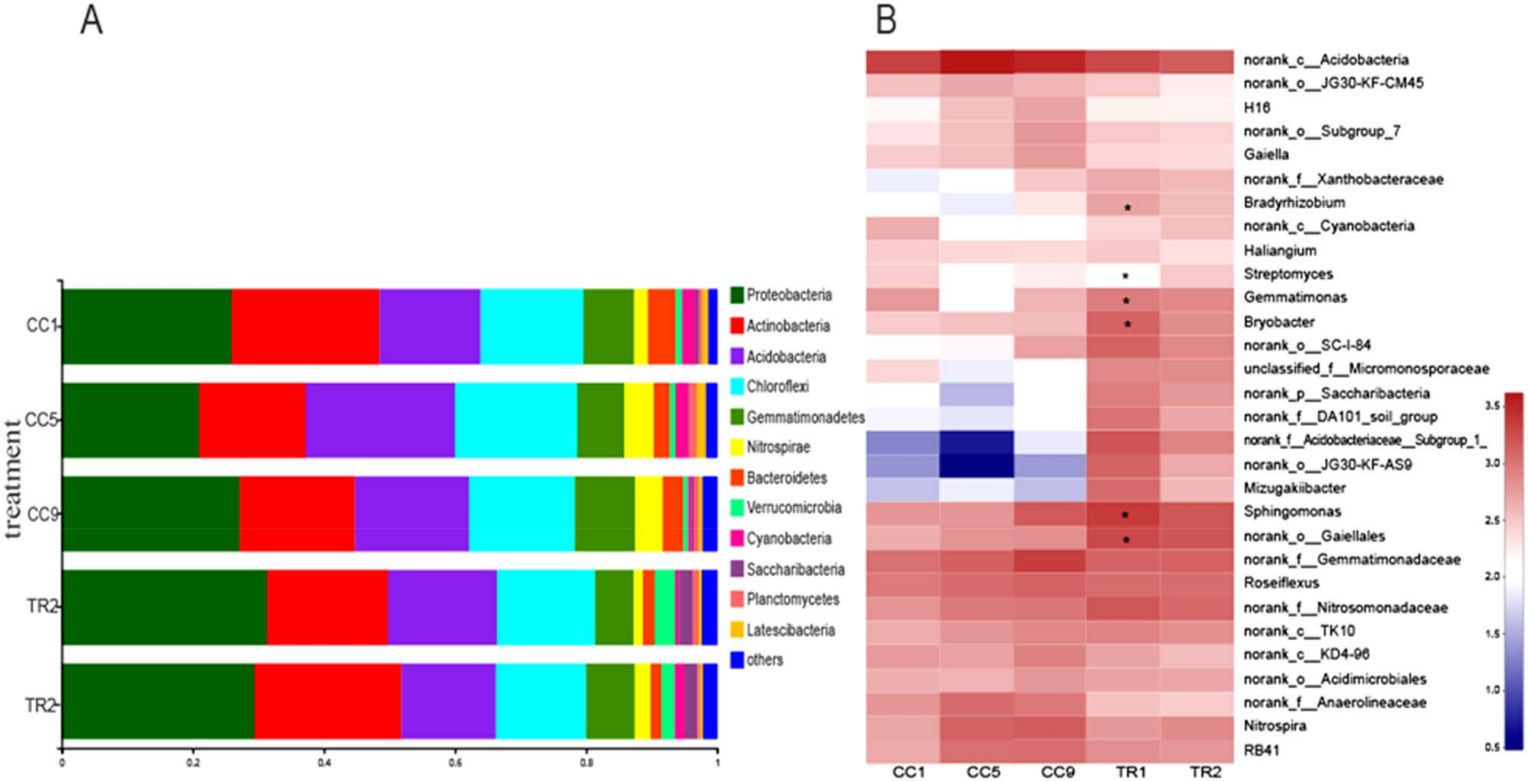
Bacterial clustering accumulation map and distribution heatmap in pepper rhizosphere soil samples at the phylum (**A**) and genus (**B**) levels. *0.01 < *P* ≤ 0.05. CC1, CC5 and CC9, represent the plots where pepper had been continuously planted for 1, 5 and 9 years, respectively, and TR1 and TR2 represent CC9 plots in which the MHT1134 biocontrol fermentation broth had been applied 1 and 2 years in advance, respectively.

At the phylum level, the species abundance analysis of the five soil samples showed that the abundance of Actinobacteria in the soil decreased gradually as the planting years increased (CC1 > CC5 > CC9), whereas the abundance of Actinobacteria in the soil increased significantly after the application of MHT1134 fermentation broth (CC9 < TR1 < TR2). The proportion increased from 17.56% (CC9) to 22.44% (TR2). On the contrary, the abundance of Acidobacteria in the pepper rhizosphere soil increased along with the planting year. However, in TR2, the abundance of Acidobacteria significantly decreased. The proportion of Acidobacteria decreased from 17.45 to 14.47% (Fig. 5A). At the genus level, the abundance levels of *Phingomonas*, *Streptomyces*, *Bryobacter*, *Mizugakiibacte* and *Gemmatimonas* increased significantly after the application of the MHT1134 fermentation broth (Fig. 5B).

### Effects of MHT1134 on the relationship between soil environmental factors and microbial community structure

**Figure 6:**
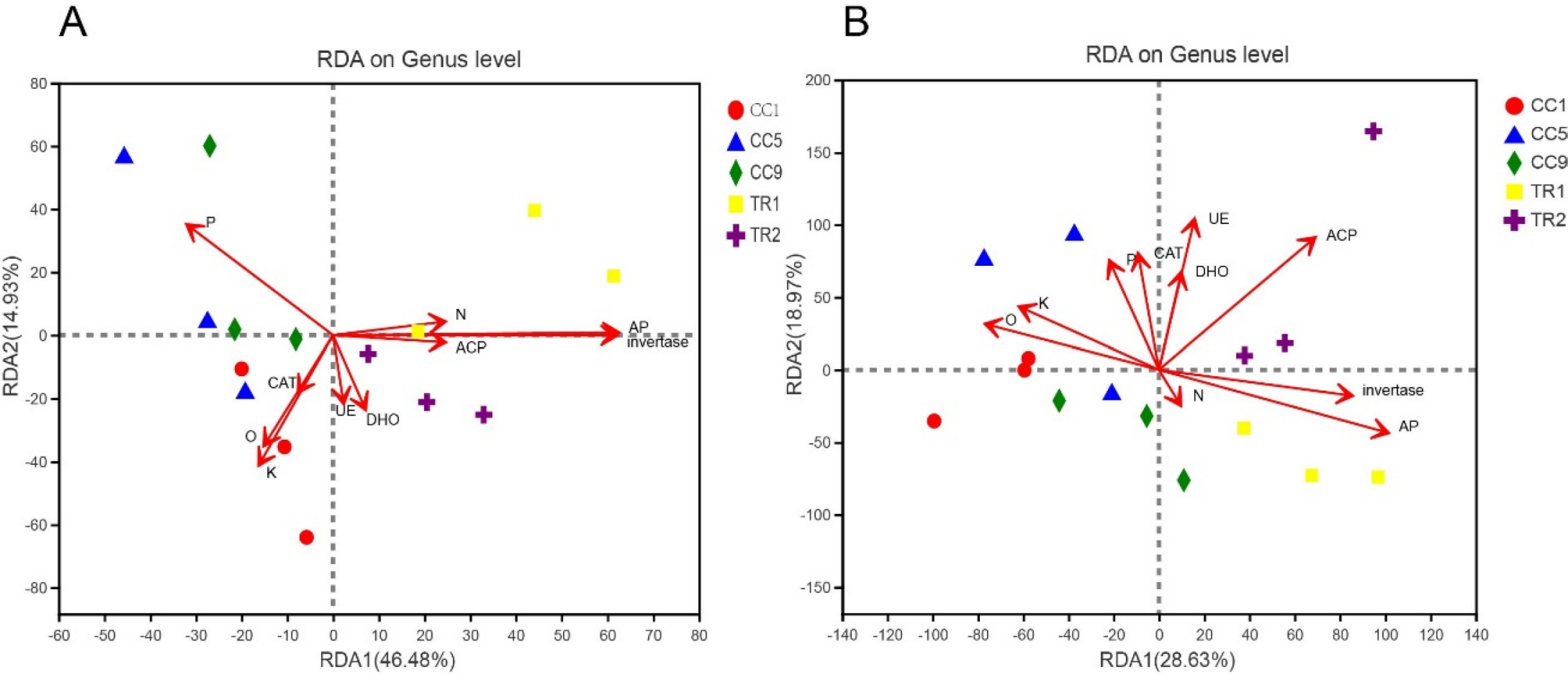
Compositions of pepper soil bacterial (**A**) and fungal (**B**) communities, and analyses of soil physical and chemical characteristics and enzyme activities using Redundancy Analysis (RDA), which can reflect the samples and environmental factors on the same two-dimensional ranking graph, so as to intuitively see the relationship between sample distribution and environmental factors). The RDA mapping data came from soil samples of the five continuous pepper cropping systems. Different coloured points or shapes in the figures represent sample groups from different environments or conditions. Red arrows indicate quantitative environmental factors. The lengths of the arrows represent the interpreted degree of influence of the environmental factors on species data. The included angles between environmental factor arrows represent positive and negative correlations (acute angle, positive correlation; obtuse angle, negative correlation; right angle, no correlation). The distance from the projection point to the origin represents the relative influence of environmental factors on the sample community distribution. *P* available phosphorus, *K* available potassium, *TN* total nitrogen, *O* organic matter, *UE* urease, *DHO* dehydrogenase; invertase, *AP* acid phosphatase, *CAT* catalase, *ACP* acid protease.

According to the redundancy analysis of the community soil bacterial and fungal genera and soil environmental factors, the contribution of bacterial community composition was 14.93% and 46.48% in principal coordinates one and two, respectively, of the Fig. 5A. In the Fig. 5B, fungal community composition contributed 18.97% and 28.63% to principal coordinates one and two, respectively. The bacterial and fungal community compositions of the five soil samples were each located in four quadrants, and the three replicates of each treatment were similar, which indicated that each treatment had good repeatability. Soil enzyme activities (phosphoacidic enzyme, dehydrogenase, urease and invertase) and physicochemical characteristics (total nitrogen, available phosphorus, available potassium and organic matter) had great effects on the bacterial community composition of the five pepper soil samples. Among the physical and chemical factors, available phosphorus had the greatest effect. Acid phosphatase, invertase and acid protease activities, as well as soil total nitrogen content, were positively correlated with soil bacterial community after the strain MHT1134 biocontrol application (Fig. 6A). Soil enzyme activities (urease, dehydrogenase, acid phosphatase, catalase, sucrase and acid protease) and physical and chemical characteristics (available phosphorus, available potassium and organic matter) had great effects on the fungal community composition of the five pepper soil samples. Among them, acid phosphatase radiation was the greatest, having the strongest effect on the pepper fungal community, and among the physical and chemical factors, effective sulphur had no obvious increasing effect on the soil fungal community composition (Fig. 6B).

## Discussion

In recent years, soil-borne diseases have become more common owing to continuous cropping practices and improper applications of pesticides and fertilizers. They have resulted in great agricultural production losses. The occurrences of soil-borne diseases are mainly correlated with an imbalance in the soil microbial community structure and the deterioration of the ecological environment^26^. The microbial diversity and uniform structure of crop rhizosphere soil decreases in plots in which soil-borne diseases occur. Deng et al.^27^ found in a comparison of the diversity of bacterial rhizosphere communities between healthy and diseased banana plants that the latter had less bacterial diversity. Xiao et al.^28^ compared the bacterial community diversity levels in the rhizosphere soils of anthrax-infected and healthy strawberry using high-throughput sequencing and found that the richness and diversity of the former were lower than the latter. He et al.^29^ found that the diversity and richness of bacteria and fungi in the soil during the peak of a watermelon *Fusarium* wilt disease incidence decreased compared with in the non-diseased control. Li et al.^30^ studied the changes in microbial community structure and diversity of cucumber rhizosphere soil in a greenhouse at 1, 3, 5 and 7 years after planting and found that the relative abundance of most bacterial genera increased slightly in the 3rd year and then decreased gradually, while the community diversity of soil samples under continuous cropping for 7 years was the lowest. Moreover, as the planting years increased, the soil bacterial function showed a downward trend.

In this study, we also analyzed the changes of microbial community structure in fields with different continuous cropping years. We found that with the increase of planting years, the diversity and abundance of fungi in pepper rhizosphere soil decreased. For bacteria, as the planting years increased, the diversity and richness of bacteria in the pepper soil also decreased. It is worth noting that the abundance of some pathogens related genera in fungi increased with the increase of planting years. For example, we found that the abundance of *Fusarium* fungi in the soil increased, as did the abundance levels of *Gibellulopsis*, *Giberella* and *Pseudallescheria*. There are many species of *Fusarium* that cause wilt on many crops. Some species in the genus *Gibellulopsis* are responsible for causing sugar beet^31^, spinach^32^ and potato^33,34^ *Verticillium* wilt. *Gibberella zeae* of the *Gibberella* genus is the pathogen responsible for wheat scab^35^. Some species of *Pseudallescheria* are alkaline tolerant. For example, Wang et al*.*^36^ reported isolating the alkali-tolerant fungus *Pseudallescheria* sp. JSM-2 from alkaline papermaking wastewater. As the continuous cropping time increases, the soil gradually becomes salinised^37^, and the abundance levels of alkaline-tolerant fungi may also increase. In addition, the abundance of Acidobacteria increased along with the planting years. Jones et al.^38^ reported that Acidobacteria are usually more prevalent in acidic environments. Therefore, it can be inferred that the increased abundance of Acidobacteria after continuous pepper cultivation indicates that this cultivation system affected the acidity and alkalinity of the soil, which further affected the fungal community structure, especially increasing the abundance of some pathogenic fungi. This indicates that some harmful microorganisms in the soil will become more abundant as the continuous cropping time increases.

Biological control is a popular green and safe measure for the prevention and control of soil-borne diseases. Its principle is to use biocontrol microorganisms or their metabolites to inhibit the reproduction and growth of pathogenic microorganisms and restore the soil microbiological ecosystem to a healthy and balanced state^39^. Inoculations of microorganisms can influence, even temporarily, the native microbial communities^40^. In this study, the characteristics of microbial communities in different continuous cropping field soils were analyzed, and biocontrol strain MHT1134 was applied to field with the longest continuous cropping time to determine the response of microbial communities and enzyme activities in continuous cropping soils. On the one hand, it is generally clear that prolonged continuous planting time leads to a great reduction in the diversity and density of soil microbes, and the abundance levels of some harmful microorganisms increases. On the other hand, in field with biocontrol MHT1134 application 1 years, soil samples had significantly increased Simpson and Sobs indexes, but the Shannon index was not significantly increased. After 2 years of continuous treatment, the Shannon index increased significantly, while the values of the other indexes slightly decreased. We speculated that the dominant species in the community was influenced by the massive colonisation of strain MHT1134 after the first application year. After continuous application, the soil ecology re-adjusted, and the soil fungal diversity level continued to increase. Moreover, the effect on the bacterial community structure in the soil was also very obvious. After the continuous application of strain MHT1134, the Simpson index increased significantly, and the dominance of bacteria in the soil increased. This is consistent with reports by Bonanomi et al.^41^ and Cordier et al.^42^. In general, the application of the biocontrol fungus MHT1134 improved the diversity and richness of microorganisms in the pepper rhizosphere soil and, to a large extent, prevented the decreasing trend in species richness caused by continuous cropping.

In addition, we found that the application of strain MHT1134 to continuous cropping fields effectively improved the microecology of the soil. On the one hand, the abundances of the fungal genera *Gibellulopsis*, *Gibberella* and *Pseudallescheria* decreased significantly, while the abundances of *Trichoderma*, *Chaetomium* and *Mortierella* increased significantly. Many varieties of *Trichoderma* are excellent sources of biocontrol bacteria for plant diseases^43,44^, and many species of *Chaetomium* also have good biocontrol effects on soil-borne plant diseases^45^. Thus, the application of strain MHT1134 increased the number of beneficial fungi in the pepper rhizosphere soil and reduced the number of harmful fungi. On the other hand, we also noticed that biocontrol strain MHT1134 had a good adjust effect on the bacterial community structure in soil. Du et al.^46^ reported that *Bryobacter* species can promote soil carbon cycling, and *Sphingomonas* is a rich new microbial resource with strong degradative functions that has great application potential in environmental protection. Interestingly, the abundance of *Sphingomonas*, *Bryobacter* and *Streptomyces* was significantly increased after the application of strain MHT1134 to the soil. And we know that there are many species of *Streptomyces* can produce antibiotic metabolites. For example, *Streptomyces* MMHS020 strain can produce caramine, ergosterol and astrosporin in soil, which can produce antibacterial activity against a variety of harmful microorganisms in soil, and promote the increase of the abundance of beneficial indicator microorganism such as *Bacillus subtilis* and *Trichoderma*^47^. This can create a virtuous circle to some extent. Therefore, we hypothesized that MHT1134 could not only increase the number of beneficial microorganisms in pepper rhizosphere soil, but also improve the microbial community structure in soil to achieve benign interaction with pathogens and host plants.

Additionally, in this study, the contents of organic matter and alkali-hydrolyzable nitrogen in soil decreased significantly after 9 years of continuous cropping. We determined that the biocontrol *Trichoderma* MHT1134 strain had a positive effect on the physical and chemical properties, as well as enzyme activities, of the soil. The alkali-hydrolysable nitrogen, organic matter, available phosphorus and available potassium contents in the soil increased after the application of strain MHT1134. This is consistent with studies that have been reported. Yin et al.^48^ reported that after applying biocontrol agents to the soil, the damage to the continuous cropping soil was repaired, to some extent, and the physical and chemical properties of the soil improved. Huang et al.^49^ applied biocontrol agents to plots in which banana *Fusarium* wilt occurred and found that the numbers of banana rhizosphere bacteria and Actinomycetes significantly increased, as did the soil organic matter, organic carbon, total nitrogen and rapidly available potassium contents.

Plant protection is achieved through enzymatic catalytic activities. Acid proteases, acid phosphatases and invertases are all hydrolases, which are involved in the hydrolysis and lysis of the molecular bonds of various compounds in soil. Acid proteases are involved in the transformation of nitrogen-containing compounds in soil, and hydrolysates are also a nitrogen source for plants. Acid phosphatase catalyses the conversion of organic phosphorus in soil to inorganic phosphorus for plant absorption. Invertase plays an important role in increasing soluble nutrients in soil, and its activity is also correlated with the number of microorganisms and rate of soil respiration. The higher the soil fertility, the higher the invertase activity. In this experiment, after the application of MHT1134, soil enzyme activity levels and physicochemical properties improved to varying degrees. The application of strain MHT1134 effectively improved the rhizosphere soil environment, enhanced the enzyme activities in the soil, and then promoted the mass reproduction of microorganisms, which is conducive to the absorption of nutrients from the soil and enhances the disease resistance of the plants.

In general, the biocontrol *Trichoderma* MHT1134 strain can reduce the disease incidence and regulate the soil microecological structure. Long-term applications effectively alleviate the harmful effects of continuous pepper cropping on the soil; consequently, it is a biocontrol resource worth developing and applying.
